# The Impact of Increased Food Availability on Reproduction in a Long-Distance Migratory Songbird: Implications for Environmental Change?

**DOI:** 10.1371/journal.pone.0111180

**Published:** 2014-10-21

**Authors:** Adam M. Seward, Colin M. Beale, Lucy Gilbert, T. Hefin Jones, Robert J. Thomas

**Affiliations:** 1 Cardiff School of Biosciences, Cardiff University, Cardiff, United Kingdom; 2 James Hutton Institute, Aberdeen, United Kingdom; University of Sydney, Australia

## Abstract

Many populations of migratory songbirds are declining or shifting in distribution. This is likely due to environmental changes that alter factors such as food availability that may have an impact on survival and/or breeding success. We tested the impact of experimentally supplemented food on the breeding success over three years of northern wheatears (*Oenanthe oenanthe*), a species in decline over much of Europe. The number of offspring fledged over the season was higher for food-supplemented birds than for control birds. The mechanisms for this effect were that food supplementation advanced breeding date, which, together with increased resources, allowed further breeding attempts. While food supplementation did not increase the clutch size, hatching success or number of chicks fledged per breeding attempt, it did increase chick size in one year of the study. The increased breeding success was greater for males than females; males could attempt to rear simultaneous broods with multiple females as well as attempting second broods, whereas females could only increase their breeding effort via second broods. Multiple brooding is rare in the study population, but this study demonstrates the potential for changes in food availability to affect wheatear breeding productivity, primarily via phenotypic flexibility in the number of breeding attempts. Our results have implications for our understanding of how wheatears may respond to natural changes in food availability due to climate changes or changes in habitat management.

## Introduction

Many animal populations are limited by food availability [1], which can itself be influenced by variation in climate, habitat quality and competition. For example, temperature and rainfall have strong effects on migrant bird populations by changing the abundance and phenology of their invertebrate food supply [2]–[5]. The availability of invertebrates to birds may also be affected by temperature and rainfall via changes in activity of invertebrates and birds or in foraging efficiency of birds [6]. This may affect the ability of insectivorous birds to obtain sufficient energy reserves for reproduction or to provide adequate food for their young [2]. Food availability may constrain the reproductive output of migrant birds by limiting the number or quality of offspring fledged in individual nesting attempts, or by limiting the number of nesting attempts during each breeding season [7], [8].

Many long distance migratory birds travel to temperate zones to take advantage of seasonal peaks in food availability for breeding. Many Palaearctic songbird populations are declining [9]; this may be due to changes in breeding productivity and/or survival rates. The underlying causes are unknown but possibilities include environmental changes in the breeding and/or wintering areas that affect the abundance of food. Successful breeding and migration both demand large quantities of food and so these stages of the annual cycle may be particularly food-limited. Food supplementation of resident and short-distance migratory songbird species usually advances laying date [10], [11]. The impacts of food supplementation on clutch size and fledgling production have been more varied, with some studies reporting increases [12]–[14], others showing no effect [15]–[17] and at least one even finding reductions in productivity [18]. Very few studies have addressed experimentally the impacts of changes in food availability on breeding productivity of long-distance migrant songbirds [19], [20] and these have focused on Nearctic-Neotropical migrants.

Here, we examine the impact of an experimental manipulation of food availability on the breeding productivity of an Old World long-distance migrant songbird, the northern wheatear (*Oenanthe oenanthe*, henceforth “wheatear”). This species is in decline over much of Europe, including the UK [21], largely attributed to changes in habitat management that alters food availability [22]. We test the impact of changing food availability on reproductive success and identify the mechanisms by determining which aspects of reproductive performance and timing are most sensitive to changes in food availability. This has implications for the potential impacts of natural changes in food supply, for example due to climate or habitat changes, on wheatear populations. Our experimental design was not intended to mirror directly the changes in food availability expected under any particular climate change scenario or land-use change. Rather, by supplementing food across whole breeding seasons, we aimed to identify which aspects of the bird's breeding performance are currently limited by food availability and thus are phenotypically flexible to climate- and habitat-linked alterations in food supply.

There is evidence that wheatear breeding success may be constrained by habitat characteristics linked to food availability. For example, wheatear territory quality (largely determined by vegetation height, which affects wheatear foraging success) appears to be more important than individual quality in determining reproductive success [23]–[25]. Wheatears holding territories with experimentally-shortened vegetation rear young much more successfully than those in which the vegetation grows taller [25]. Adult survival rates are also higher in wheatears breeding in territories with short field layers [26]. Food supplementation of wheatears in a breeding area led to increased survival rates of both adults and fledged juveniles [27], indicating that determinants of fitness are food-limited. Examining the impact of food-supplementation on breeding performance will test whether the food availability also limits the investment of adult wheatears in egg laying and brood provisioning.

The key aim of this study was to use a food supplementation experiment to test the hypothesis that food availability impacts on breeding success in wheatears. Furthermore, we explored the mechanisms for this effect by examining which of the following key aspects of the breeding cycle were most limited by food availability: timing of breeding, clutch size, egg size, hatching success, chick size, number of fledglings per nesting attempt and rates of multiple brooding.

## Materials and Methods

### Ethics statement

All of the field experiments and animal protocols were conducted with the permission of the Fair Isle Bird Observatory Trustees and the National Trust for Scotland. Bird ringing was licensed by the British Trust for Ornithology. This study did not involve protected species and the birds were not collected. The sampling methods were non-invasive and are described fully in the Methods section. No individuals were sacrificed or harmed in any way. The vertebrate work involved provision of supplementary food to wild birds in their natural habitat. The birds' natural food supply was available to all birds throughout the study. The study was thus was non-invasive and indeed the food supplementation experiment was expected to be beneficial to those individuals (and their offspring) that received the supplementary food. We therefore did not seek approval from an Institutional Animal Care and Use Committee.

### Study location and species

The study was conducted on Fair Isle (59°32′N, 1°39′W), a ca. 1,000 ha island lying north-east of the Scottish mainland, UK. Breeding wheatears arrive on Fair Isle between the beginning of April and mid-May. Nests are located in holes in the ground, under rocks or in dry stone walls. First clutch egg-laying begins in early May and continues into June. Clutch size ranges from 4 to 8 eggs; incubation, by the female, lasts about 13 days (range 10–18 days [28]). Both parents provision the chicks. Chicks fledge after approximately 15 days. The parents continue to feed fledglings until they become independent, about two weeks after fledging.

Adult wheatears were captured with spring traps (www.moudry.cz, model SB30). Plumage features were used to sex and age captured birds as fledged in the previous year (young), before the previous year (old) or fledged before the current year (unknown if young or old) [29]. All males, but only a minority of females, could be aged as young or old. All captured birds were measured (maximum wing chord to 1 mm), weighed to 0.1 g, and fitted with a numbered metal ring and a unique combination of plastic colour rings to enable individual identification in the field. Nestlings were ringed when approximately 7 days old.

### Feeding experiment

Prey availability for wheatears was increased by providing mealworms in plastic bowls placed on the ground. During 2008–2010, feeders were available from territory establishment in late April/early May, and filled with at least 30 g of mealworms (mean ± SD  = 37.8±2.7 g, n = 20 mealworm samples). Feeders were refilled on at least five days in each week, until autumn departure of all breeding birds and their offspring (August/early September). Mean (± SD) first egg laying date of fed pairs was 17.1±5.1 days after each feeder was set up (N = 24 fed territories with this information available Sample sizes of supplementary fed (treatment) and unfed (control) wheatear pairs were: 2008 – 15 fed, 14 control; 2009 – 23 fed, 22 control; 2010 – 27 fed, 27 control.

As there are age-related differences in arrival date and breeding success of wheatears [23], [30], a daily standard study site route was walked from mid-April until the end of May, and newly established breeding pairs were selected alternately as fed and control (i.e. unfed) pairs. A pair was selected if behavioural signs of pair establishment were observed. In this way, fed and control pairs were stratified both spatially and with respect to arrival date. This procedure also meant that supplementary feeding only began after territory establishment, thus avoiding the potentially confounding situation of the highest quality individuals establishing territories around feeders, to the exclusion of lower quality individuals.

One feeder was located in the estimated centre of each food-supplemented territory. Direct observation and footage from small video cameras (Sony Handycam, model DCR-SR32) confirmed the identities of the wheatears using the feeders. At least three recording sessions of at least 1 hour each – made on different days and at different times during daylight – were viewed per feeder, but viewing sessions were extended to 4 hours if neither or only one of a pair had attended the feeder during the initial period. During this video monitoring, none of the adult wheatears from control pairs were ever recorded taking mealworms from any of the feeders in any year. Wheatears from supplementary fed pairs were sometimes recorded taking mealworms from other feeders outside their own territory. 88.3% of feeding visits in a random sample of 20 recording sessions were made by the target pair. The remaining visits were by European starlings (*Sturnus vulgaris*).

Wire mesh cages permitted wheatears but not European starlings to access feeding bowls through a small hole cut at the bottom or via a hinged weighted walkway that swung shut when the heavier starlings attempted to enter, swinging back open again when the starling stepped off the platform. Fed pairs started using feeders within a few days of them being set up. To let wheatears get used to attending feeders, one month generally passed between positioning feeders and deploying cages. In each year, however, some wheatear pairs (n = 5, 6 and 2 in 2008, 2009 and 2010, respectively) that had initially attended feeders stopped using them once the cages were positioned. Such pairs were excluded from some analyses, as explained below. The feeders that were used by the target wheatears became depleted of mealworms between top-ups and as approximately 88% of visits were by the target pair, we estimate that, on average, at least 23.5 g of mealworms – approximately 88% of the minimum of 27.0 g available (37.8 g per feed ×5 days (minimum)/7 days  = 27.0 g) – were taken by each target pair per day.

### Reproductive parameters

Nests were found by observing the parents going to and from nest holes. On finding each nest, its status was recorded as (i) being built, containing (ii) eggs or (iii) chicks. Nest contents were subsequently checked every other day and the number of any dead chicks or un-hatched eggs recorded. Laying date, clutch size, egg volume, hatching date, hatching success, chick wing length and number of fledglings were recorded (Table S1). Egg volume was recorded because of the possibility that supplementary feeding may influence female investment in egg production, with potential carry-over effects on chick size [31].

### Data analysis

It is important to measure the degree of correlation between pairs of reproductive parameters; strong correlation between a pair of reproductive parameters suggests that an extraneous factor impacting the earliest-occurring variable of the pair will affect the later-occurring variable indirectly. A correlation matrix revealed only weak levels of co-variation between most pairs of reproductive parameters (Table 1). The analysis focuses on the effects of food supplementation on each of the reproductive success parameters outlined in Table 2. Year, adult age, and other individual characteristics may also affect reproductive success. Furthermore, aspects of breeding parameters early on in a breeding attempt (e.g. breeding date, clutch size) may influence later measures of breeding success (e.g. chick size, number of juveniles). To investigate these effects, a series of models were fitted for each response variable using the statistical package R, version 3.0.3 [32]. Where appropriate (see below and Table 2), we used general linear models, linear mixed models (fitted by maximum likelihood) and generalised linear mixed models (fitted by maximum likelihood). Mixed models were fitted using the lme4 package [33] within R. Intercept-only models (null models) were included within each set of candidate models. Treatment (fed or control), year and the interaction of treatment and year were included amongst the candidate models of each response variable unless the response was measured in only a single year. Hatching dates were used as the measure of breeding timing because more data was available for hatching than for laying dates and the two variables were highly correlated (R = 0.91, P<0.001, N = 22). As well as analysing hatching date as a breeding parameter itself, it was included as a fixed effect (and the interaction of treatment and hatching date) in all other analyses except for nest survival. For nest survival, the date on which the nest was found (and the interaction of treatment and date found) was used instead. Values of hatching date or date nest found were standardized by subtracting the mean and dividing by the standard deviation. Male age (young (yearling) or old (2+ years old)) was included, as well as the interaction of treatment and male age, amongst candidate models of hatching date to account for the possible impact of male age on arrival times and therefore breeding date and territory quality. For other analyses, hatching date was used instead because territory quality has been shown to be more important for reproductive success than individual characteristics [23]–[25]. For chick size, chick age (7 days-old or 8 days old) was included in all models (except for a null model) as a controlling factor and number of chicks was considered in some of the candidate models. All subsets of the full models in each case have been considered (except for chick size, where chick age was kept in all models as a necessary control). The full lists of candidate models are provided in Tables S2–S11. The strength of support for competing candidate models was assessed using AIC corrected for small sample size (AICc [34]) and AICc weights (*w*AICc). The plausible models were defined as those with ΔAIC*_i_* ≤2. For each reproductive parameter, we carried out multi-model inference to derive model-averaged parameter estimates and confidence intervals of fixed effects included in plausible models, based on *w*AICc of each model *i* [34]. Model averaging of main effects excluded models in which those main effects were also included in interaction terms. Model ranking by AICc and model averaging of parameter estimates was carried out with the R package AICcmodavg [35].

**Table 1:** Correlation matrix of reproductive parameters investigated in this study.

	HD	Clutch size	Egg size	Hatched eggs	Chick wing	First fledglings	Attempts	Fledglings per season
HD	1.00							
Clutch size	−0.22	1.00						
Egg size	−0.22	−0.19	1.00					
Hatched eggs	−0.23	0.59	0.33	1.00				
Chick wing	0.14	−0.13	0.08	−0.16	1.00			
First fledglings	−0.03	0.29	0.24	0.62	0.25	1.00		
Attempts	−0.35	0.18	0.08	0.22	−0.23	0.11	1.00	
Fledglings per season	−0.26	0.38	0.28	0.51	−0.05	0.66	0.73	1.00

Values are Pearson's correlation coefficients between pairs of reproductive parameters per territory per year, with male identity used to represent territory.

HD  =  hatching date of first clutch, Clutch size  =  number of eggs in first clutch, Egg size  =  mean egg volume per first clutch, Hatched eggs  =  number of eggs in first clutch that hatched, Chick wing  =  mean maximum wing chord of chicks per nest, First fledglings  =  Number of young that fledged from first nesting attempts, Attempts  =  number of breeding attempts per male per season, Fledglings per season  =  number of young fledged per male per season.

**Table 2:** Description of the fixed effects and model types used to analyse each reproductive parameter.

Breeding parameter	Fixed effects	Random effects	Model type	Notes
Chicks fledged nesting attempt^−1^	trt, HD, yr, trt × HD, trt × yr	Female ID	Poisson GLMM	First clutches only. Excluded: failures due to predation or rabbit (*Oryctolagus cuniculus*) disturbance, re-laid clutches. For polygynous males, only earliest clutch included.
Chicks fledged male^−1^ season^−1^	trt, HD, yr, trt × HD, trt × yr	Male ID	Poisson GLMM	Only marked individuals included.
Chicks fledged female^−1^ season^−1^	trt, HD, yr, trt × HD, trt × yr	Female ID	Poisson GLMM	Only marked individuals included.
Clutch size	trt, HD, yr, trt × HD, trt × yr	-	Poisson GLM	2009 and 2010 only. Random effect not needed because there was no pseudoreplication.
Egg volume	trt, HD, trt × HD	Female ID	LME	Only measured in 2010. Ln transformed to achieve normality for analysis.
Hatching date	trt, maleage, yr, trt × maleage, trt × yr	Male ID	LME	Earliest clutch for each male only.
Hatching success *(probability of each* *egg hatching)*	trt, HD, yr, trt × HD, trt × yr	Female ID	Binomial GLMM	First broods only. For clutches with total hatching failure, HD calculated from first egg laying date, assuming 1 egg laid per day and average incubation for study population (12.46).
Chick maximum wing chord	trt, chicks, HD, yr, chickage, trt × HD, trt × yr	Female ID	LME	Only measured in 2009 and 2010. First broods only. 7- and 8-day-old chicks. Chick age included in all candidate models except null model.
Nest survival	trt, found, yr, trt × found, trt × yr	-	Binomial GLM	For each nest, modelled daily probability of nest failure based on days active and days failed (Mayfield [36]). One datum sampled per individual female.
Breeding attempts male^−1^ season^−1^	trt, HD, trt × HD, trt × yr	-	Binomial GLM	Re-lays not included as additional attempts. Marked individuals only.

Fixed effects (in order of appearance in table). trt  =  treatment (fed or control), HD  =  standardized hatching date of earliest brood for that individual or pair, yr  =  year, maleage  =  male age (young (yearling) or old (2+)), chicks  =  number of nestlings alive at time of measurement, chickage  =  age of chicks (7 or 8 days old), found  =  standardized date nest found. Male ID/Female ID  =  individual identity of male/female. GLMM  =  Generalized linear mixed model fit by maximum likelihood, GLM  =  General linear model, LME  =  Linear mixed model fit by maximum likelihood.

Female identity (ID) was included where appropriate as a random effect in mixed models to account for multiple broods of individuals between and across years. Male ID was used as the random effect for the analyses of young fledged per male per breeding season and number of breeding attempts per male per breeding season. Male ID was also used as the random effect for the analysis of hatching date because male age was included as a factor (we had less data on female age). Male and female ID were never included together, female ID being nested within male ID. Several candidate models of nest survival failed to converge when fitted with random effects. For the analysis of this parameter, pseudoreplication was avoided by randomly selecting one datum per individual by the *sample* procedure in R (dataset reduced from 110 nests to 99 nests) and the models were fitted with general linear models instead. For the analysis of multiple brooding by males, initial modelling for random effects did not allow for year effects because of the lack of contrasts in multiple brooding available between all of the factors treatment, hatching date and year. Modelling with random effects without year provided a standard deviation of 0 for the random effect of male ID, indicating that multiple brooding did not depend on individual identity. To allow for year effects to be included in the candidate models without discarding data, models of multiple brooding were conducted with general linear models. Unringed individuals were included within some analyses. The number of unringed individuals was relatively high in 2008 (10 males/7 females of 22 pairs), but fewer in 2009 and 2010 (5 males/7 females of 40 pairs in 2009; 5 males/5 females of 48 pairs in 2010). Thus there may have been some pseudoreplication of unringed breeders between years, although only about 50% of unringed adult birds are expected to return the following year [17]. Fed pairs that stopped using feeders when cages were deployed were included in analyses of clutch size and egg volume, as they were still being fed during those stages of the breeding cycle. For all other analyses, these pairs were excluded from the dataset.

The total number of chicks fledged across the whole breeding season was obtained for individual parents instead of pairs (new pairs could form after failed nesting attempts and males could have simultaneous broods with multiple females). Individuals with failed broods were included in the analysis because feeding treatment may influence decisions about re-laying following nest failure. As there were no cases of multiple broods within the control group of females, we did not use linear modelling to test for an effect of food supplementation on multiple brooding by females. Instead, we used a Fisher Exact Test to test the significance of the treatment effect. Details of the models run for each breeding parameter are listed in Table 2.

## Results

144 wheatear nests were found during 2008–2010. 129 of these were first clutches, three were re-lays after first clutch failure, eight were simultaneous clutches and four were second clutches. Of these nests, the identity of both parents was known for 102, the identity of the male only was known for 15, the identity of the female only was known for 19 and the identity of neither parent was known for 8 pairs. The most direct measure of reproductive success is the number of fledglings produced. These results are described first. Other reproductive parameters are then examined to investigate the mechanisms by which the food availability increase may influence reproductive output.

### Fledging success

#### Chicks fledged per nesting attempt

There was no effect of food supplementation on the number of chicks fledged per nesting attempt amongst first broods (mean ± SE  = 5.26±0.23 for fed broods, 5.04±0.22 for control broods, Table S2). The best fitting model included only year as a factor, while the null model was the only other plausible model (Table 3, Models 1–2). Model-averaged parameter estimates indicated that the number of chicks fledged per nest was highest in 2010 and lowest in 2008 (Table 4).

**Table 3:** Models fitted to different reproductive parameters.

Reproductive parameter	Model ID	Fixed effects	Random effects	K	ΔAICc*_i_*	*w*AICc*_i_*
Chicks fledged nesting attempt^−1^	1	yr	Female ID	4	0.000	0.285
	2	none	Female ID	2	0.309	0.244
Chicks fledged male^−1^ season^−1^	3	trt × HD, yr	Male ID	8	0.000	0.594
Chicks fledged female^−1^ season^−1^	4	none	Female ID	2	0.000	0.326
	5	trt	Female ID	3	1.003	0.197
	6	yr	Female ID	4	1.999	0.120
Clutch size	7	none	-	1	0.000	0.462
	8	yr	-	2	1.631	0.204
Log egg volume	9	none	Female ID	3	0.000	0.379
	10	HD	Female ID	4	0.336	0.321
Hatching date	11	trt, yr	Male ID	6	0.000	0.358
	12	trt, yr, maleage	Male ID	7	0.761	0.244
Hatching success	13	HD	Female ID	3	0.000	0.335
	14	none	Female ID	2	1.079	0.195
Chick maximum wing chord	15	trt × yr, chickage	Female ID	7	0.000	0.288
Nest survival	16	none	-	2	0.000	0.327
	17	trt	-	2	1.323	0.169
	18	found	-	2	1.889	0.127
Breeding attempts male^−1^ season^−1^	19	trt, HD	-	3	0.000	0.392
	20	trt × HD	-	4	0.920	0.247

AICc is the corrected Akaike's Information Criterion, ΔAICci is the difference in AICc between model *i* and the best model and *w*AICc*_i_* is the Akaike weight. Plausible models (ΔAICc*_i_* ≤2) are presented; see Tables S2–S14 for the full sets of candidate models. Interactions are indicated by × and include all lower order additive terms as well.

Fixed effects (in order of appearance in table). yr  =  year, none  =  intercept-only model, trt  =  treatment (fed or control), HD  =  standardized hatching date, maleage  =  male age (young (yearling) or old (2+)), chickage  =  age of chicks, found  =  standardized date nest found.

**Table 4:** Model-averaged parameter estimates (estimates of fixed effects included in models with ΔAICc*_i_* ≤2 with contributions to average weighted by *w*AICc*_i_* of model), unconditional standard errors and 95% confidence intervals.

Reproductive parameter	Fixed effect	Estimate	SE	95% CI	
				Lower	Upper
Chicks fledged nesting attempt^−1^	2009 vs. 2008	0.214	0.177	−0.134	0.562
	2010 vs. 2008	0.330	0.165	0.007	0.654
Chicks fledged male^−1^ season^−1^	Fed vs. Control × Hatching date	−0.275	0.102	−0.475	−0.076
	2009 vs. 2008	−0.118	0.179	−0.469	0.233
	2010 vs. 2008	0.171	0.171	−0.164	0.505
Chicks fledged female^−1^ season^−1^	Fed vs. Control	0.099	0.097	−0.091	0.289
	2009 vs. 2008	−0.118	0.179	−0.469	0.233
	2010 vs. 2008	0.958	0.845	−0.698	2.615
Clutch size	2010 vs. 2009	0.089	0.123	−0.152	0.330
Log egg volume	Hatching date	−0.016	0.012	−0.040	0.007
Hatching date	Fed vs. Control	−2.953	1.107	−5.124	−0.783
	Young vs. Old male	1.452	1.175	−0.850	3.755
	2009 vs. 2008	−2.159	1.875	−5.834	1.516
	2010 vs. 2009	1.459	1.890	−2.245	5.163
Hatching success	Hatching date	−0.796	0.449	−1.677	0.084
Chick maximum wing chord	8 days old vs. 7 days old	5.604	1.309	3.038	8.170
	Fed vs. Control × 2010 vs. 2009	−7.463	2.834	−13.018	−1.908
Nest survival	Fed vs. Control	0.826	0.806	−0.754	2.407
	Date found	−0.129	0.358	−0.831	0.573
Breeding attempts male^−1^ season^−1^	Fed vs. Control	2.574	1.146	0.327	4.820
	Hatching date	−1.083	0.487	−2.036	−0.129
	Fed vs. Control × Hatching date	−1.173	1.099	−3.326	0.980

Interactions are indicated by x.

Hatching date (1 =  1^st^ May) and date found (date nest discovered; 1 =  1^st^ May) were standardized before being input in models as fixed factors.

#### Chicks fledged per breeding season

**Figure 1:**
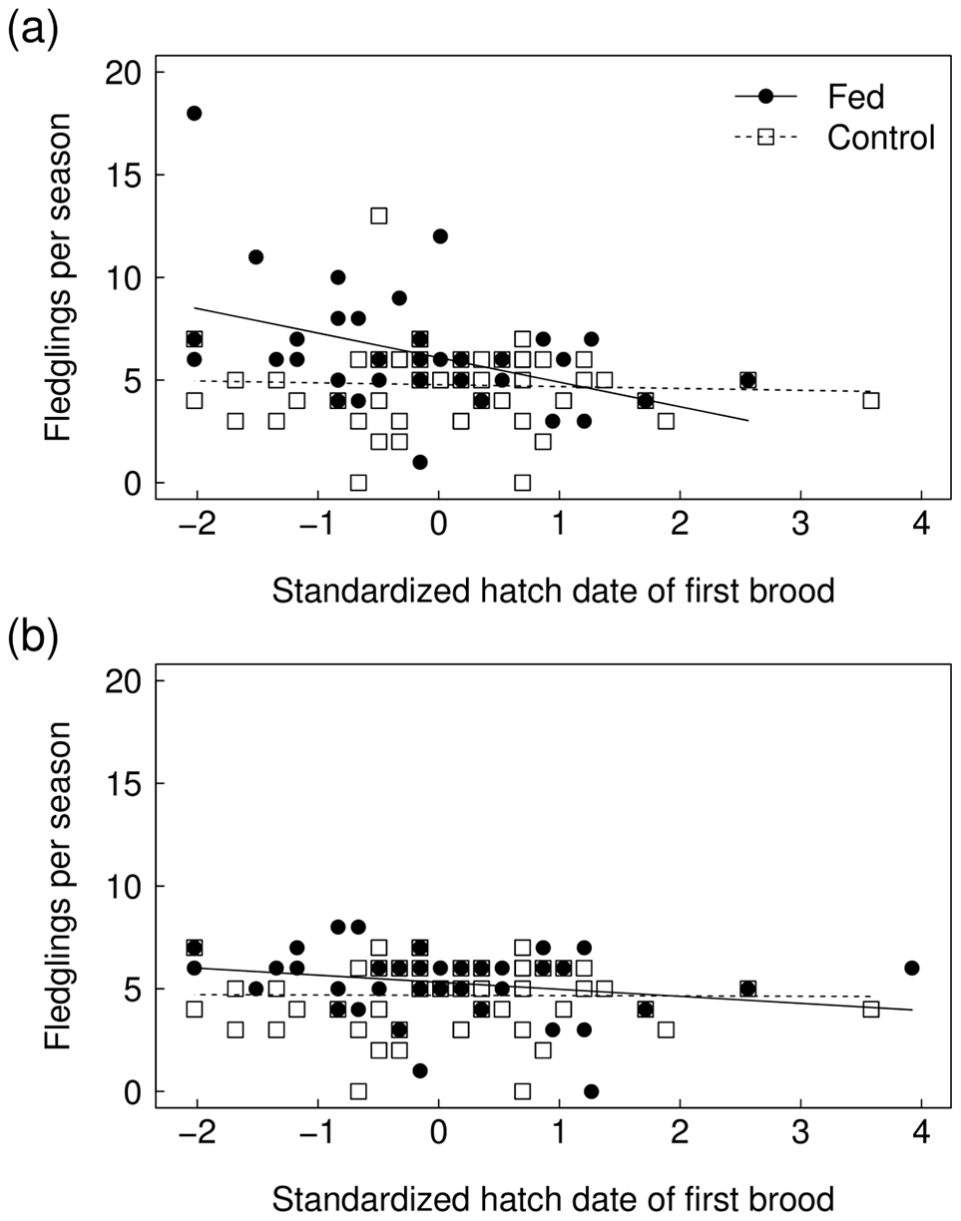
The relationship between standardized first brood hatching date and number of juveniles fledged across the season by food-supplemented and control male (a) and female (b) wheatears. Lines of best fit produced by linear models are shown to aid interpretation.

More young were fledged per breeding season by fed males (mean ± SE  = 6.32±0.48 fledglings male^−1^, N = 37) than by control males (5.14±0.29 fledglings male^−1^, N = 43). Only one plausible model was identified, including the treatment × standardized hatching date interaction and year (Table 3, Model 3; see Table S3 for the AICc model fits of all candidate models). Fed males fledged more young over the course of a season the earlier they started breeding, while there was no such relationship amongst control males (Figure 1a; Table 4).

More young were fledged per breeding season by fed females (5.37±0.26 fledglings female^−1^, N = 41) than by control females (4.84±0.25 fledglings female^−1^, N = 44). The best model was, however, the null model (Table 3, Model 4). The other two plausible models included treatment alone and year alone (Table 3, Models 5 and 6; see Table S4 for the AICc model fits of all candidate models). Model-averaged parameter estimates showed that the effect of treatment and year on young fledged per season by females were only weak (Figure 1b, Table 4).

### Clutch size

Mean clutch size was 6.26±0.10 eggs (range 4–8, N = 57). Two plausible models were identified: the null model and a model including year (Table 3, Models 7 and 8; see Table S5 for the AICc model fits of all candidate models). Model averaged parameter estimates indicated that there was only weak annual variation in clutch size (Table 4).

### Egg volume

Mean egg volume was 2761±14.42 mm^3^ (N = 258). Two plausible models were identified: the null model and a model including standardized hatching date (Table 3, Models 9 and 10; see Table S6 for the AICc model fits of all candidate models). Model averaged parameter estimates indicated that egg volume decreased weakly with standardized hatching date (Table 4).

### Hatching date

**Figure 2:**
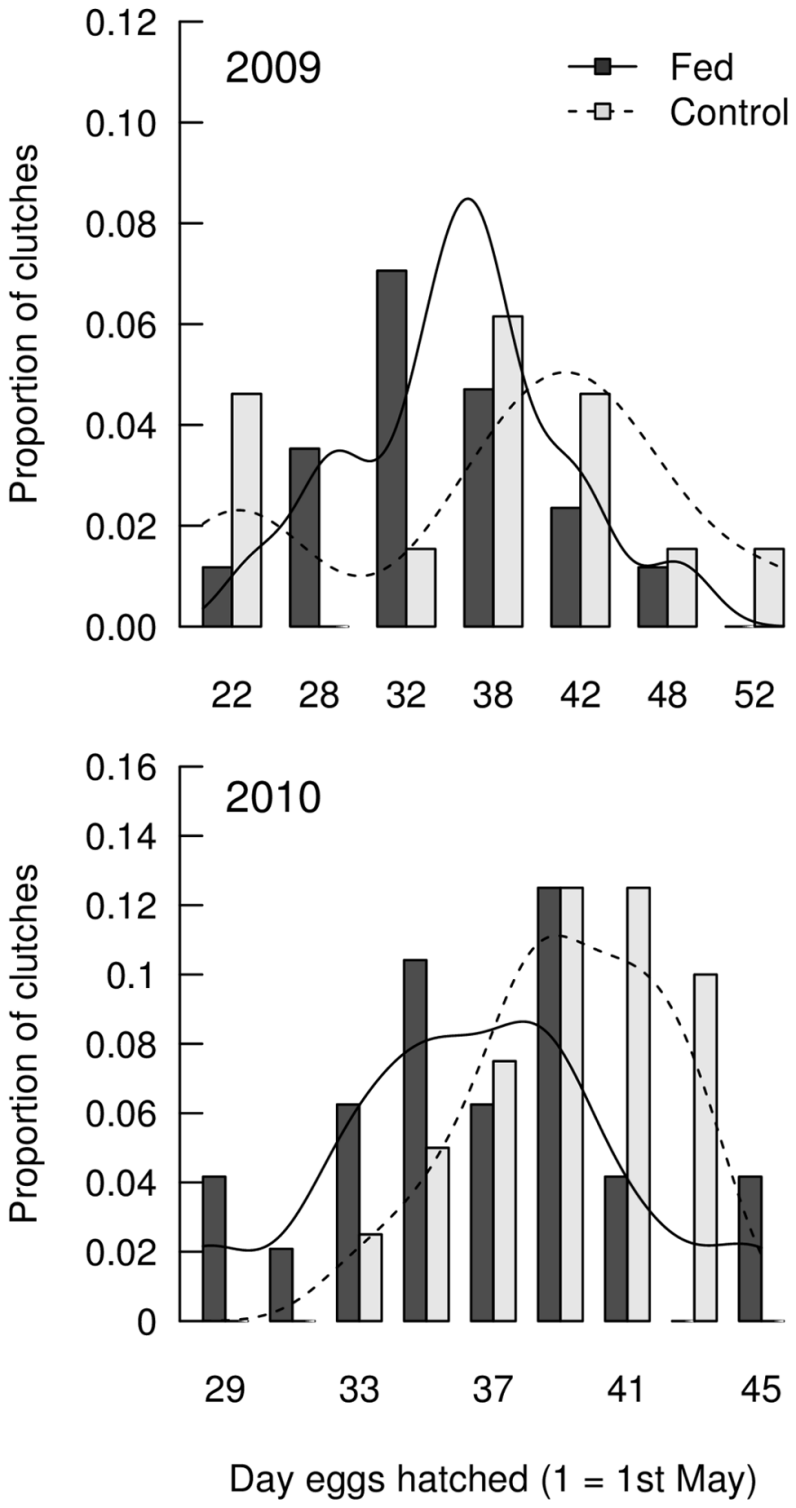
Distribution of dates of hatching of first clutches in 2009 and 2010 according to treatment. Density estimation curves shown to aid interpretation (solid  =  fed, dashed  =  control).

Hatching dates of first clutches ranged from 31^st^ May to 22^nd^ June in 2008 (N = 9), 20^th^ May to 22^nd^ June in 2009 (N = 33) and from 28^th^ May to 15^th^ June in 2010 (N = 46). Hatching dates in 2009 and 2010 only were included in the analysis because of the sparseness of data for 2008. Two plausible models of hatching date were identified, both of which included treatment and year and one included male age (Table 3, Models 11 and 12; see Table S7 for the AICc model fits of all candidate models). Model averaged parameter estimates indicated that fed males hatched their first clutches 2.95 days earlier than control males (Table 4, Figure 2). There were weak trends for clutches of yearling males to hatch later than clutches of 2+ year old males (Table 4). There was also weak annual variation in hatching dates (Table 4).

### Hatching success

Of 60 first nesting attempts with known clutch size, four completely failed to hatch and 18 had partial hatching failure (at least one egg did not hatch). Two plausible models of hatching success were identified: one with standardized hatching date alone and the other was the null model (Table 3, Models 13 and 14; see Table S8 for the AICc model fits of all candidate models). Model averaged parameter estimates indicated that there was a weak inverse relationship between standardized hatching date and hatching success (Table 4).

### Chick size

**Figure 3:**
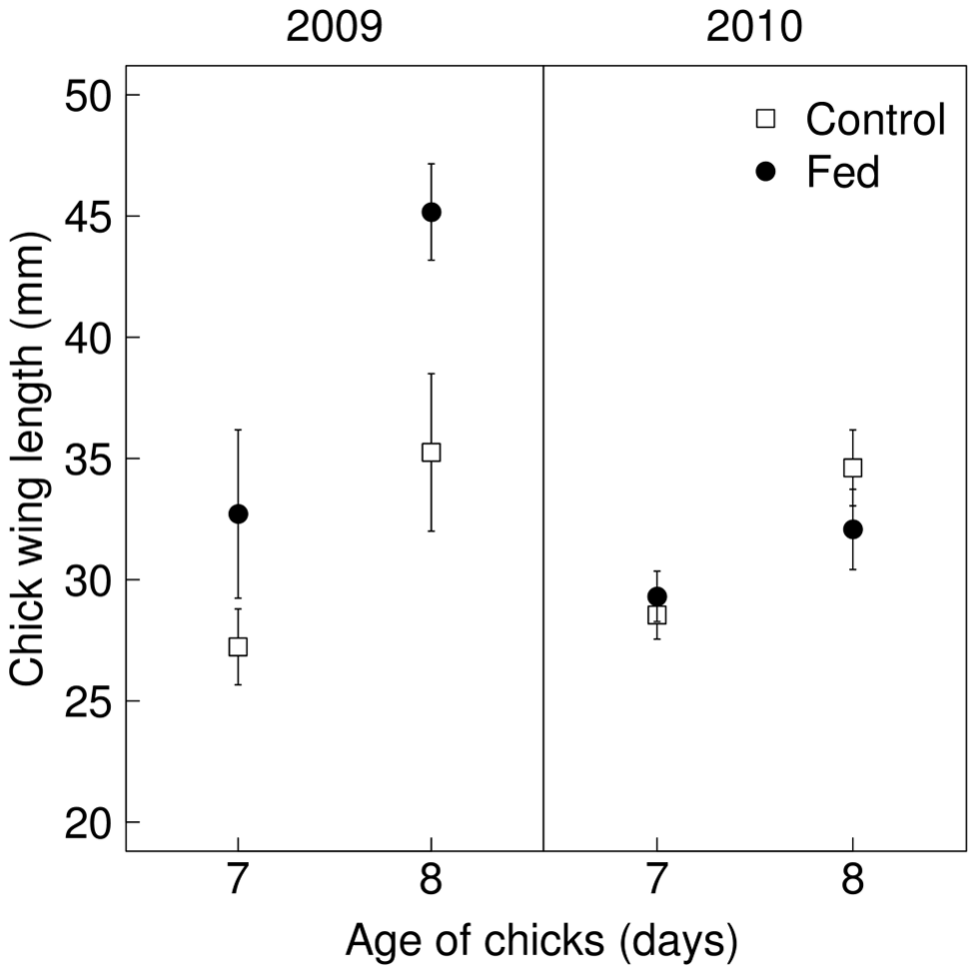
The effect of food supplementation on chick wing length. Variation in wing length (mean ±95% confidence limits) in relation to age for fed and control chicks in 2009 and 2010.

Mean maximum wing chord (the measure of chick size) was 28.95±0.34 mm for 7-day-old chicks (range 13 to 41, N = 266) and 34.69±0.63 mm for 8-day-old chicks (range 16 to 48, N = 70). The interaction of treatment and year was included in the only plausible model of chick size (Table 3, Model 15; see Table S9 for the AICc model fits of all candidate models). Model averaged parameter estimates indicated that treatment had a greater effect on chick maximum wing chord in 2009 than in 2010 (Table 4). In fact, a plot of maximum wing chord by year shows that fed chicks were larger than control chicks in 2009 but that there was no difference between them in 2010 (Figure 3).

### Nest survival

Out of 36 nests of fed parents, 34 were successful (≥1 chick fledged) and 54 of 63 nests of control parents were successful. Mean Mayfield daily nest survival rates [36] were 99.5±0.4% for nests of fed parents and 97.2±1.2% for nests of control parents. There were three plausible models of nest survival: the null model, one with treatment alone and one with standardized nest finding date alone (Table 3, Models 16 to 18; see Table S10 for the AICc model fits of all candidate models). The confidence intervals for the effects of treatment and standardized nest finding date indicated little statistical support for either factor (Table 4).

### Probability of multiple brooding per breeding season

#### Males

Nine of 37 fed and one of 43 control males had multiple broods. Two plausible models of probability of multiple brooding were identified: one including treatment and standardized hatching date and the other including the interaction of treatment and standardized hatching date (Table 3, Models 19 and 20; see Table S11 for the AICc model fits of all candidate models). Model averaged parameter estimates indicated that fed males were more likely to have multiple broods than were control males and that standardized hatching date had a significant inverse effect on the probability of multiple brooding (Table 4). The interaction of treatment and standardized hatching date was not well supported by the data, having confidence intervals that overlapped zero by a wide margin (Table 4).

#### Females

Four of 37 fed females had second broods (these were all consecutive broods with the same male). None of the 41 unfed females had second broods. Fed females were significantly more likely to have multiple broods than control females (Fisher's Exact Test *P* = 0.046).

## Discussion

This study tested the impact of experimentally manipulated food availability on the reproductive performance of the northern wheatear, a long-distance migratory insectivorous bird, with implications for the potential effects of climate- and land-use-driven changes in food in the breeding areas. The experimental increase in food availability increased the annual reproductive output of the wheatears breeding on Fair Isle by increasing the frequency of pairs raising second broods and males raising broods with more than one female (polygyny). The experimental increase in food availability had no detectable effect on clutch size or the number of fledglings produced from first broods.

As well as leading to an increase in the production of fledglings produced over the breeding season, food supplementation also advanced hatching date of first broods and increased chick quality (as measured by body size). Specifically, increased food availability led to an advance in hatching date of first broods by approximately 3 days, an increase in the wing length of chicks measured at 7 and 8 days of age in 2009 (but not in 2010) (which could either represent a larger fledging size or more rapid growth towards an unaltered fledging size).

The advance in hatching dates of first broods of food-supplemented wheatears relative to controls indicates that the timing of breeding was constrained by natural food availability on Fair Isle. Food supplementation within a territory was initiated only once a pair was seen to have formed by their behaviour. This was necessary to avoid a situation where birds arriving on Fair Isle in the spring chose those territories with supplementary food, which could have led to the feeding treatment being distributed unevenly with respect to individual quality and arrival dates and confounded the experiment. Clutches of fed pairs were initiated approximately 17 days after feeders within their territories were deployed. The result of designing the experiment in this way means that food supplementation likely occurred over only part of the adult female developmental phase of reproduction, which occurs before egg laying [37]. Despite this, we observed a 3-day advance in hatching dates (highly correlated with laying dates) of fed wheatears relative to unsupplemented controls. Advances in breeding date from food supplementation at the breeding site are also widely reported in resident and short-distance migratory songbirds [10], [11]. The extent of advance in breeding date in response to increased food was similar to our study for some of these species (e.g. 2 days for jackdaws *Corvus monedula* and 5 days for great tits *Parus major*) [14], [38] but much greater in others, such as 18 days in song sparrows (*Melospiza melodi*) [15]. The timing of initiation of supplemental feeding relative to laying dates may influence the extent of advance in laying dates, but the link is not clear; in the three examples above, experimental provisioning began much earlier in the jackdaw study than in the great tit or song sparrow studies.

The earlier date of first clutches induced by food supplementation may have contributed to an increased reproductive output by increasing the time available for females to lay a second clutch following fledging of their first. Additional breeding attempts were very rare by control pairs, however, even by those that began breeding early, suggesting that the increase in the number of breeding attempts was likely to depend on food availability in combination with early breeding, and not just on early breeding *per se*. Wheatears that initiated their first brood earlier and had access to supplemental food were more likely to have second broods (and, in the case of males, have simultaneous broods). A higher proportion of pairs that initiated a first brood subsequently initiated second broods in 2009 (6.5%) than in 2010 (1.9%), which is consistent with the earlier start to breeding in 2009 (hatch date in 2009 was 8 days earlier than in 2010). The proportion of males initiating simultaneous broods was, however, higher in 2010 (9.6%) than in 2009 (4.3%), but simultaneous brooding will be less time-limited than second brooding. Consistent with our findings, experimental and natural changes in food availability led to changes in numbers of breeding attempts in black-throated blue warblers (*Dendroica caerulescens*) [8], [39] and song sparrows [15]. In support of our finding that earlier breeding alone does not increase rates of multiple brooding, food-supplementation of resident breeding birds during pre-laying and laying stages only resulted in advanced laying dates but no impact on number of clutches initiated [40], [41]. In an observational study of wheatears [42], earlier breeding was associated with greater fledging success of first broods and with a higher probability of second brooding, but in the absence of food manipulation it is not certain whether food availability (as breeding timing is likely related to territory quality) underpinned these results. In a long-term Swedish study, older male wheatears had higher reproductive success than yearling males, probably because older birds arrive on breeding grounds earlier, allowing them to commence breeding sooner as well as potentially gain the best territories [24], [25]. In our experimental study, high quality early-arriving males with supplementary food may be able to expend more energy on defending larger territories and attracting additional mates compared to lower quality late-arriving males that may be more constrained in their territory choice.

The low rates of total nest failure in this study are in stark contrast with other studies of wheatears. Only about 8% of first clutches in the current study failed to produce any fledglings, while total failure rates of about 41%, 30% and 21% were recorded in East Anglia [42] and two studies in Sweden [22], [28], respectively. It is therefore possible that increases in food availability have a greater effect on the reproductive parameters measured on Fair Isle than among wheatears living in areas where predation risk is a greater determinant of breeding success. This would be an interesting avenue of future research.

The supplemented food in our study also resulted in increased chick (and therefore probably fledgling) quality, as measured by chick size at age 7–8 days. These results are consistent with those of natural and experimental food reduction, which led to decreased nestling growth rates of black-throated blue warblers [39]. The magnitude of the effect in our study did, however, vary between years, suggesting that food availability is not always equally limiting to chick growth.

Although food supplementation had measurable effects on hatching date, the number of breeding attempts and chick growth, other reproductive parameters appeared to be unaffected. There was no difference in clutch size or hatching success of first broods, which explains why food-supplementation also did not increase the number of chicks fledged from first broods. It is possible that our sample size was not large enough to detect an effect. In support of our results, however, food supplementation had no effect on the number of fledglings from first broods in black-throated blue warblers [8], while clutch size was higher in food-supplemented pairs in only five of 14 species (comprising non-migratory and short-distance migrant species) reviewed by Arcese and Smith [15].

The present study provides evidence that the number of breeding attempts that can be fitted into each breeding season is currently limited both by food availability, and by the date of initiation of the first brood. Our food supplementation was uniformly high across the whole breeding season, yet high altitude and high latitude moorland habitats typically have short growing/breeding seasons, with highly peaked food availability [43]. Phenological changes associated with climate change are, indeed, already resulting in earlier spring arrivals of migratory songbirds. The timing of autumn departures are also advancing, however, leading to a shift in the breeding season with no increase in its duration [44]–[46]. The variation between years in the frequency of second broods in our study suggests that other factors (e.g. weather and availability of key prey taxa), as well as overall food availability, may affect multiple brooding. As well as environmental conditions at the breeding grounds, breeding date of migratory birds depends on spring arrival date and arrival body condition, both of which are affected by environmental factors at the wintering grounds and migration routes [5], [47], [48]. Breeding close to the wintering grounds, resident and short-distance migrants may be less time-constrained in their response to environmental change than long-distance migrants [49]. Temperature changes may also have direct impacts on bird reproduction that may have consequences for duration of the breeding season. For example, great tits kept at higher temperatures began laying at the same time as controls but terminated laying, regressed their testes and started post-breeding moult earlier, despite food being provided ad libitum [50]. To understand the implications of our finding that food availability may affect fledgling production in wheatears via changes in breeding attempts, it is important to understand how climate change will affect the timing and shape of food peaks, food abundance and the direct impact on wheatear behaviour and physiology of factors such as temperature.

This study has shown that food supplementation increased fitness of northern wheatears by providing resources needed for second broods. In addition, while no additional chicks were fledged from first broods, the chicks were larger which may aid post-fledging survival [27] and increase fitness as an adult. This has clear implications for the impact of changes in food availability due to environmental change, although caution is always required in extrapolating results to other populations, species or even to other years [43]. There may also be independent, direct effects of environmental variables such as temperature and rainfall on breeding parameters [51], [52]. Much of the literature on the impacts of climate change on birds has focussed on the issue of mismatches between timing of breeding and the timing of peaks in food availability [53]–[55]. By increasing food availability uniformly across the whole breeding season, we have been unable to address the impact of changes in the timing of peaks in the food supply of wheatears. Instead, our experiment measured the degree of phenotypic plasticity in a range of breeding parameters, revealing the extent to which individual birds can respond instantly to changes in environmental conditions. If the range of climate (and thus food) variability that the birds' phenotypic plasticity encompasses is exceeded, then there will be selective pressure for evolutionary change.

## Supporting Information

Table S1 **Reproductive parameters recorded for breeding wheatears.**
(DOCX)

Table S2 **Model comparisons for number of fledglings per first nesting attempt.** First broods only. Excludes total failures due to predation and fed pairs that stopped using feeders when starling exclusion cages were placed over them. Random effect is Female ID. K is the number of parameters in the model. AICc is the corrected Akaike's Information Criterion, ΔAICc*_i_* is the difference in AICc between model *_i_* and the best model and *w*AICc*_i_* is the AICc weight of the model. Interactions are indicated by × and include all lower order terms as well (e.g. trt × HD represents trt + HD + trt × HD).
(DOCX)

Table S3 **Model comparisons for number of juveniles fledged per male per season.** Random effect is Male ID. AICc is the corrected Akaike's Information Criterion, ΔAICc*_i_* is the difference in AICc between model *_i_* and the best model and *w*AICc*_i_* is the AICc weight of the model. Interactions are indicated by × and include all lower order terms as well (e.g. trt × HD represents trt + HD + trt × HD).
(DOCX)

Table S4 **Model comparisons for number of juveniles fledged per female per season.** Random effect is female ID. AICc is the corrected Akaike's Information Criterion, ΔAICc*_i_* is the difference in AICc between model *_i_* and the best model and *w*AICc*_i_* is the AICc weight of the model. Interactions are indicated by × and include all lower order terms as well (e.g. trt × HD represents trt + HD + trt × HD).
(DOCX)

Table S5 **Linear model comparisons for clutch size in 2009 and 2010.** AICc is the corrected Akaike's Information Criterion, ΔAICc*_i_* is the difference in AICc between model *_i_* and the best model and *w*AICc*_i_* is the AICc weight of the model. Interactions are indicated by × and include all lower order terms as well (e.g. trt × HD represents trt + HD + trt × HD).
(DOCX)

Table S6 **Model comparisons for egg volume in 2010.** Random effect is female ID. AICc is the corrected Akaike's Information Criterion, ΔAICc*_i_* is the difference in AICc between model *_i_* and the best model and *w*AICc*_i_* is the AICc weight of the model. Interactions are indicated by × and include all lower order terms as well (e.g. trt × HD represents trt + HD + trt × HD).
(DOCX)

Table S7 **Model comparisons for hatching date.** Random effect is Male ID. AICc is the corrected Akaike's Information Criterion, ΔAICc*_i_* is the difference in AICc between model *_i_* and the best model and *w*AICc*_i_* is the AICc weight of the model. Interactions are indicated by × and include all lower order terms as well (e.g. trt × maleage represents trt + maleage + trt × maleage).
(DOCX)

Table S8 **Model comparisons for hatching success.** Random effect is female ID. AICc is the corrected Akaike's Information Criterion, ΔAICc*_i_* is the difference in AICc between model *_i_* and the best model and *w*AICc*_i_* is the AICc weight of the model. Interactions are indicated by × and include all lower order terms as well (e.g. trt × age represents trt + HD + trt × HD).
(DOCX)

Table S9 **Model comparisons for maximum wing chord of 7- and 8-day-old chicks in 2009 and 2010.** Random effect is Female ID. AICc is the corrected Akaike's Information Criterion, ΔAICc*_i_* is the difference in AICc between model *_i_* and the best model and *w*AICc*_i_* is the AICc weight of the model. Interactions are indicated by × and include all lower order terms as well (e.g. trt × HD represents trt + HD + trt × HD).
(DOCX)

Table S10 **Model comparisons for first brood nest survival.** All models have Male ID as the random factor. Main effects were included for all interactions. AICc is the corrected Akaike's Information Criterion, ΔAICc*_i_* is the difference in AICc between model *_i_* and the best model and *w*AICc*_i_* is the AICc weight of the model. Interactions are indicated by × and include all lower order terms as well (e.g. trt × found represents trt + found + trt × found).
(DOCX)

Table S11 **Model comparisons for probability of multiple brooding by males per breeding season.** Breeding attempts included first clutches, second clutches (after a successful first clutch) and simultaneous clutches (i.e. polygyny) but did not include re-lays. For the analysis, males either did (1) or did not (0) have multiple broods. AICc is the corrected Akaike's Information Criterion, ΔAICc*_i_* is the difference in AICc between model *_i_* and the best model and *w*AICc*_i_* is the AICc weight of the model. Interactions are indicated by × and include all lower order terms as well (e.g. trt × HD represents trt + HD + trt × HD).
(DOCX)
