# Supplementary material for: The Impact of Increased Food Availability on Reproduction in a Long-Distance Migratory Songbird: Implications for Environmental Change?
Source: PLoS One. 2014 Oct 21;9(10):e111180. doi: 10.1371/journal.pone.0111180 (PMC4205087; doi:10.1371/journal.pone.0111180)
Supplement: Table S1 — Reproductive parameters recorded for breeding wheatears. (DOCX) [file pone.0111180.s001.docx]

**Table S1. Reproductive parameters recorded for breeding wheatears.**

| **Parameter** | **Description** |
| --- | --- |
| Identity of parents | The colour ring combinations of the birds involved in incubation and provisioning was recorded. |
| Laying date | The day on which the first egg of a clutch was laid (Day 1 = 1^st^ May). Only estimated for clutches of known size. Estimated by back-dating from date last egg laid or hatching date minus average incubation duration for clutches in this study (12.46 days), assuming one egg was laid per day [1]. |
| Clutch size | The total number of eggs in the completed clutch (first clutches only). Nest contents were not directly visible as the nests were always built in burrows or in rock piles, and so the eggs were counted by touch. These counts proved to be 100% accurate when eggs were taken out of the nest for measuring. Clutches were considered to be complete once no new eggs were found on successive visits and the eggs were warm (and therefore being incubated). |
| Egg volume | In 2010, maximum length and maximum width of the eggs of all accessible clutches were measured (to 0.1 mm) using plastic callipers. Egg volume was then calculated following Hoyt [2]. |
| Incubation duration | The number of days between the last egg being laid (which is the first day of regular incubation of the clutch) and the first egg hatching. |
| Hatching date | The day that the first egg or eggs hatched. If all the chicks had already hatched when the nest was found, hatching date was estimated based on development of the chicks, using data from chicks of known age in the study population. |
| Hatching success | The number of chicks that hatched calculated from the clutch size, minus the number of unhatched eggs found in the nest. |
| Chick size | Chick maximum wing chord (to 1 mm) and body mass (to 0.1 g) were measured at between 5 and 8 days old (usually at 7 days) in 2009 and 2010, at the same time as being fitted with a numbered metal ring. Time of measurement of chicks was random with respect to feeding treatment. |
| Number of fledglings | The number of chicks surviving to leave the nest. Fledglings were either counted or calculated by subtracting the number of dead chicks found in the nest cavity after fledging (once fledglings were observed and/or parents were giving intense warning calls from 15 days after the hatching date) from the number of live chicks that were present in the nest at 5-8 days old [3]. Although this method does not account for predation of nestlings between ringing and fledging, partial nest predation of wheatears is very rare (less than 1% of successful nests in a detailed study in Sweden [3], and so should not have a major effect on the results. |
| Nest survival | After locating active nests, they were checked every 2 or 3 days and recorded as active, failed (no chicks fledged) or successful (if at least one chick was fledged). Successful breeding attempts were defined as nests where fledglings were observed and/or parents gave intense warning calls from 15 days after the hatching date [3]. |
| Multiple brooding probability | For individuals with successful first broods, the presence or absence of a second breeding attempt was recorded. A second breeding attempt was recorded if at least 1 egg was laid. A separate analysis was carried out, individual males and individual females, since additional broods might be attempted by members of the original pair subsequently or (in the case of males) simultaneously pairing with other partners for a second breeding attempt. |

References

1. Conder P (1989) The Wheatear. London: Christopher Helm. 328 p.
2. Hoyt DF (1979) Practical methods of estimating volume and fresh weight of bird eggs. The Auk 96: 73-77.
3. Arlt D, Forslund P, Jeppsson T, Pärt T (2008) Habitat-specific population growth of a farmland bird. PloS ONE 3: e3006.
