# Supplementary material for: The Impact of Increased Food Availability on Reproduction in a Long-Distance Migratory Songbird: Implications for Environmental Change?
Source: PLoS One. 2014 Oct 21;9(10):e111180. doi: 10.1371/journal.pone.0111180 (PMC4205087; doi:10.1371/journal.pone.0111180)
Supplement: Table S2 — Model comparisons for number of fledglings per first nesting attempt. First broods only. Excludes total failures due to predation and fed pairs that stopped using feeders when starling exclusion cages were placed over them. Random effect is Female ID. K is the number of parameters in the model. AICc is the corrected Akaike's Information Criterion, ΔAICci is the difference in AICc between model i and the best model and wAICci is the AICc weight of the model. Interactions are indicated by × and include all lower order terms as well (e.g. trt × HD represents trt + HD + trt × HD). (DOCX) [file pone.0111180.s002.docx]

**Table S2. Model comparisons for number of fledglings per first nesting attempt.** First broods only. Excludes total failures due to predation and fed pairs that stopped using feeders when starling exclusion cages were placed over them. Random effect is Female ID. K is the number of parameters in the model. AICc is the corrected Akaike’s Information Criterion, ΔAICc*_i_* is the difference in AICc between model *_i_* and the best model and *w*AICc*_i_* is the AICc weight of the model. Interactions are indicated by x and include all lower order terms as well (e.g. trt x HD represents trt + HD + trt x HD).

| **Fixed effects** | **K** | **AICc** | **ΔAICc_i_** | **wAICc_i_** | **Log-likelihood** |
| --- | --- | --- | --- | --- | --- |
| yr | 4 | 321.010 | 0.000 | 0.285 | -156.242 |
| none | 2 | 321.319 | 0.309 | 0.244 | -158.583 |
| HD, yr | 5 | 323.110 | 2.100 | 0.100 | -156.155 |
| trt, yr | 5 | 323.277 | 2.267 | 0.092 | -156.239 |
| trt | 3 | 323.301 | 2.291 | 0.091 | -158.495 |
| HD | 3 | 323.316 | 2.305 | 0.090 | -158.502 |
| trt, HD | 4 | 325.397 | 4.387 | 0.032 | -158.435 |
| trt, HD, yr | 6 | 325.445 | 4.435 | 0.031 | -156.155 |
| trt x HD | 5 | 327.212 | 6.202 | 0.013 | -158.206 |
| trt x HD, yr | 7 | 327.659 | 6.648 | 0.010 | -156.062 |
| trt x yr | 7 | 327.744 | 6.733 | 0.010 | -156.105 |
| trt x yr, HD | 8 | 330.019 | 9.009 | 0.003 | -156.009 |
| trt x HD, trt x yr | 9 | 332.104 | 11.094 | 0.001 | -155.784 |

Fixed effects: trt: treatment (fed or control), yr: year, HD: standardized hatching date, none: intercept-only model.
