# Supplementary material for: The Impact of Increased Food Availability on Reproduction in a Long-Distance Migratory Songbird: Implications for Environmental Change?
Source: PLoS One. 2014 Oct 21;9(10):e111180. doi: 10.1371/journal.pone.0111180 (PMC4205087; doi:10.1371/journal.pone.0111180)
Supplement: Table S4 — Model comparisons for number of juveniles fledged per female per season. Random effect is female ID. AICc is the corrected Akaike's Information Criterion, ΔAICci is the difference in AICc between model i and the best model and wAICci is the AICc weight of the model. Interactions are indicated by × and include all lower order terms as well (e.g. trt × HD represents trt + HD + trt × HD). (DOCX) [file pone.0111180.s004.docx]

**Table S4. Model comparisons for number of juveniles fledged per female per season.** Random effect is female ID. AICc is the corrected Akaike’s Information Criterion, ΔAICc*_i_* is the difference in AICc between model *_i_* and the best model and *w*AICc*_i_* is the AICc weight of the model. Interactions are indicated by x and include all lower order terms as well (e.g. trt x HD represents trt + HD + trt x HD).

| **Fixed effects** | **K** | **AICc** | **ΔAICci** | **wAICci** | **Log-likelihood** |
| --- | --- | --- | --- | --- | --- |
| none | 2 | 353.550 | 0.000 | 0.326 | -174.702 |
| trt | 3 | 354.553 | 1.003 | 0.197 | -174.128 |
| yr | 4 | 355.549 | 1.999 | 0.120 | -173.525 |
| HD | 3 | 355.670 | 2.119 | 0.113 | -174.687 |
| trt, HD | 4 | 356.757 | 3.206 | 0.066 | -174.128 |
| trt, yr | 5 | 357.025 | 3.475 | 0.057 | -173.133 |
| HD, yr | 5 | 357.595 | 4.044 | 0.043 | -173.418 |
| trt x HD | 5 | 358.326 | 4.775 | 0.030 | -173.783 |
| trt, HD, yr | 6 | 359.250 | 5.700 | 0.019 | -173.087 |
| trt x yr | 7 | 359.644 | 6.094 | 0.015 | -172.095 |
| trt x HD, yr | 7 | 361.060 | 7.510 | 0.008 | -172.803 |
| trt x yr, HD | 8 | 361.954 | 8.403 | 0.005 | -172.029 |
| trt x HD, trt x yr | 9 | 364.265 | 10.715 | 0.002 | -171.933 |

Fixed effects: trt: treatment (fed or control), yr: year, HD: hatching date of first brood, none: intercept-only model.
