# Supplementary material for: The Impact of Increased Food Availability on Reproduction in a Long-Distance Migratory Songbird: Implications for Environmental Change?
Source: PLoS One. 2014 Oct 21;9(10):e111180. doi: 10.1371/journal.pone.0111180 (PMC4205087; doi:10.1371/journal.pone.0111180)
Supplement: Table S5 — Linear model comparisons for clutch size in 2009 and 2010. AICc is the corrected Akaike's Information Criterion, ΔAICci is the difference in AICc between model i and the best model and wAICci is the AICc weight of the model. Interactions are indicated by × and include all lower order terms as well (e.g. trt × HD represents trt + HD + trt × HD). (DOCX) [file pone.0111180.s005.docx]

**Table S5. Linear model comparisons for clutch size in 2009 and 2010.** AICc is the corrected Akaike’s Information Criterion, ΔAICc*_i_* is the difference in AICc between model *_i_* and the best model and *w*AICc*_i_* is the AICc weight of the model. Interactions are indicated by x and include all lower order terms as well (e.g. trt x HD represents trt + HD + trt x HD).

| **Fixed effects** | **K** | **AICc** | **ΔAICci** | **wAICci** | **Log-likelihood** |
| --- | --- | --- | --- | --- | --- |
| none | 1 | 217.618 | 0.000 | 0.462 | -107.773 |
| yr | 2 | 219.249 | 1.631 | 0.204 | -107.514 |
| HD | 2 | 219.723 | 2.105 | 0.161 | -107.750 |
| HD, yr | 3 | 221.411 | 3.793 | 0.069 | -107.479 |
| trt | 3 | 221.955 | 4.337 | 0.053 | -107.751 |
| trt, yr | 4 | 223.701 | 6.083 | 0.022 | -107.466 |
| trt, HD | 4 | 224.245 | 6.627 | 0.017 | -107.738 |
| trt, HD, yr | 5 | 226.069 | 8.451 | 0.007 | -107.446 |
| trt x yr | 6 | 228.538 | 10.920 | 0.002 | -107.429 |
| trt x HD | 6 | 229.074 | 11.456 | 0.002 | -107.697 |
| trt x yr, HD | 7 | 231.088 | 13.470 | 0.001 | -107.401 |
| trt x HD, yr | 7 | 231.152 | 13.534 | 0.001 | -107.433 |
| trt x HD, trt x yr | 9 | 236.622 | 19.004 | 0.000 | -107.396 |

Fixed effects: trt: treatment (fed or control), HD: standardized hatching date, yr: year, none: intercept-only model.
