# Supplementary material for: The Impact of Increased Food Availability on Reproduction in a Long-Distance Migratory Songbird: Implications for Environmental Change?
Source: PLoS One. 2014 Oct 21;9(10):e111180. doi: 10.1371/journal.pone.0111180 (PMC4205087; doi:10.1371/journal.pone.0111180)
Supplement: Table S6 — Model comparisons for egg volume in 2010. Random effect is female ID. AICc is the corrected Akaike's Information Criterion, ΔAICci is the difference in AICc between model i and the best model and wAICci is the AICc weight of the model. Interactions are indicated by × and include all lower order terms as well (e.g. trt × HD represents trt + HD + trt × HD). (DOCX) [file pone.0111180.s006.docx]

**Table S6. Model comparisons for egg volume in 2010.** Random effect is female ID. AICc is the corrected Akaike’s Information Criterion, ΔAICc*_i_* is the difference in AICc between model *_i_* and the best model and *w*AICc*_i_* is the AICc weight of the model. Interactions are indicated by x and include all lower order terms as well (e.g. trt x HD represents trt + HD + trt x HD).

| **Fixed effects** | **K** | **AICc** | **ΔAICc_i_** | **wAICc_i_** | **Log-likelihood** |
| --- | --- | --- | --- | --- | --- |
| none | 3 | -849.075 | 0.000 | 0.379 | 427.585 |
| HD | 4 | -848.739 | 0.336 | 0.321 | 428.448 |
| trt | 4 | -847.013 | 2.062 | 0.135 | 427.586 |
| trt, HD | 5 | -846.797 | 2.279 | 0.121 | 428.517 |
| trt x HD | 6 | -844.742 | 4.334 | 0.043 | 428.538 |

Fixed effects: trt: treatment (fed or control), HD: standardized hatching date, , none: intercept-only model.
