# Supplementary material for: The Impact of Increased Food Availability on Reproduction in a Long-Distance Migratory Songbird: Implications for Environmental Change?
Source: PLoS One. 2014 Oct 21;9(10):e111180. doi: 10.1371/journal.pone.0111180 (PMC4205087; doi:10.1371/journal.pone.0111180)
Supplement: Table S7 — Model comparisons for hatching date. Random effect is Male ID. AICc is the corrected Akaike's Information Criterion, ΔAICci is the difference in AICc between model i and the best model and wAICci is the AICc weight of the model. Interactions are indicated by × and include all lower order terms as well (e.g. trt × maleage represents trt + maleage + trt × maleage). (DOCX) [file pone.0111180.s007.docx]

**Table S7. Model comparisons for hatching date.** Random effect is Male ID. AICc is the corrected Akaike’s Information Criterion, ΔAICc*_i_* is the difference in AICc between model *_i_* and the best model and *w*AICc*_i_* is the AICc weight of the model. Interactions are indicated by x and include all lower order terms as well (e.g. trt x maleage represents trt + maleage + trt x maleage).

| **Fixed effects** | **K** | **AICc** | **ΔAICci** | **wAICci** | **Log-likelihood** |
| --- | --- | --- | --- | --- | --- |
| trt, yr | 6 | 562.248 | 0.000 | 0.358 | -274.605 |
| trt, yr, maleage | 7 | 563.009 | 0.761 | 0.244 | -273.805 |
| trt x maleage, yr | 8 | 564.394 | 2.146 | 0.122 | -273.285 |
| trt | 4 | 565.925 | 3.677 | 0.057 | -278.721 |
| trt x yr | 8 | 566.275 | 4.027 | 0.048 | -274.226 |
| yr | 5 | 566.753 | 4.505 | 0.038 | -278.011 |
| trt x yr, maleage | 9 | 566.965 | 4.717 | 0.034 | -273.328 |
| trt, maleage | 5 | 567.240 | 4.992 | 0.029 | -278.254 |
| yr, maleage | 6 | 567.809 | 5.561 | 0.022 | -277.386 |
| trt x maleage | 6 | 568.115 | 5.867 | 0.019 | -277.539 |
| trt x maleage, trt x yr | 10 | 568.451 | 6.203 | 0.016 | -272.797 |
| none | 3 | 569.681 | 7.433 | 0.009 | -281.697 |
| maleage | 4 | 571.095 | 8.847 | 0.004 | -281.306 |

Fixed effects: trt: treatment (fed or control), maleage: male age (young (yearling) or old (2+)), yr: year, none: intercept-only model.
