# Supplementary material for: The Impact of Increased Food Availability on Reproduction in a Long-Distance Migratory Songbird: Implications for Environmental Change?
Source: PLoS One. 2014 Oct 21;9(10):e111180. doi: 10.1371/journal.pone.0111180 (PMC4205087; doi:10.1371/journal.pone.0111180)
Supplement: Table S8 — Model comparisons for hatching success. Random effect is female ID. AICc is the corrected Akaike's Information Criterion, ΔAICci is the difference in AICc between model i and the best model and wAICci is the AICc weight of the model. Interactions are indicated by × and include all lower order terms as well (e.g. trt × age represents trt + HD + trt × HD). (DOCX) [file pone.0111180.s008.docx]

**Table S8. Model comparisons for hatching success.** Random effect is female ID. AICc is the corrected Akaike’s Information Criterion, ΔAICc*_i_* is the difference in AICc between model *_i_* and the best model and *w*AICc*_i_* is the AICc weight of the model. Interactions are indicated by x and include all lower order terms as well (e.g. trt x age represents trt + HD + trt x HD).

| **Fixed effects** | **K** | **AICc** | **ΔAICci** | **wAICci** | **Log-likelihood** |
| --- | --- | --- | --- | --- | --- |
| HD | 3 | 137.359 | 0.000 | 0.335 | -65.465 |
| none | 2 | 138.438 | 1.079 | 0.195 | -67.114 |
| HD, yr | 4 | 139.640 | 2.281 | 0.107 | -65.457 |
| trt, HD | 4 | 139.654 | 2.295 | 0.106 | -65.463 |
| trt | 3 | 140.553 | 3.193 | 0.068 | -67.062 |
| yr | 3 | 140.640 | 3.280 | 0.065 | -67.106 |
| trt x HD | 5 | 141.899 | 4.539 | 0.035 | -65.394 |
| trt, HD, yr | 5 | 142.021 | 4.661 | 0.033 | -65.455 |
| trt, yr | 4 | 142.829 | 5.469 | 0.022 | -67.051 |
| trt x yr, HD | 6 | 143.877 | 6.518 | 0.013 | -65.146 |
| trt x HD, yr | 6 | 144.335 | 6.976 | 0.010 | -65.375 |
| trt x yr | 5 | 145.029 | 7.669 | 0.007 | -66.959 |
| trt x HD, trt x yr | 7 | 146.247 | 8.888 | 0.004 | -65.047 |

Fixed effects: trt: treatment (fed or control), yr: year, HD: standardized hatching date, none: intercept-only model.
