# Supplementary material for: The Impact of Increased Food Availability on Reproduction in a Long-Distance Migratory Songbird: Implications for Environmental Change?
Source: PLoS One. 2014 Oct 21;9(10):e111180. doi: 10.1371/journal.pone.0111180 (PMC4205087; doi:10.1371/journal.pone.0111180)
Supplement: Table S9 — Model comparisons for maximum wing chord of 7- and 8-day-old chicks in 2009 and 2010. Random effect is Female ID. AICc is the corrected Akaike's Information Criterion, ΔAICci is the difference in AICc between model i and the best model and wAICci is the AICc weight of the model. Interactions are indicated by × and include all lower order terms as well (e.g. trt × HD represents trt + HD + trt × HD). (DOCX) [file pone.0111180.s009.docx]

**Table S9. Model comparisons for maximum wing chord of 7- and 8-day-old chicks in 2009 and 2010.** Random effect is Female ID. AICc is the corrected Akaike’s Information Criterion, ΔAICc*_i_* is the difference in AICc between model *_i_* and the best model and *w*AICc*_i_* is the AICc weight of the model. Interactions are indicated by x and include all lower order terms as well (e.g. trt x HD represents trt + HD + trt x HD).

| **Fixed effects** | **K** | **AICc** | **ΔAICc_i_** | **wAICc_i_** | **Log-likelihood** |
| --- | --- | --- | --- | --- | --- |
| trt x yr, chickage | 7 | 1948.819 | 0.000 | 0.288 | -967.242 |
| trt x yr, chicks, chickage | 8 | 1950.907 | 2.089 | 0.101 | -967.238 |
| trt x yr, HD, chickage | 8 | 1950.915 | 2.096 | 0.101 | -967.241 |
| trt x chicks, trt x yr, chickage | 9 | 1951.010 | 2.191 | 0.096 | -966.234 |
| trt, chickage | 5 | 1951.444 | 2.625 | 0.078 | -970.632 |
| trt x yr, chicks, HD, chickage | 9 | 1953.016 | 4.197 | 0.035 | -967.237 |
| trt x HD, trt x yr, chickage | 9 | 1953.019 | 4.200 | 0.035 | -967.238 |
| trt x chicks, trt x yr, HD, chickage | 10 | 1953.124 | 4.305 | 0.033 | -966.230 |
| trt, HD, chickage | 6 | 1953.151 | 4.332 | 0.033 | -970.450 |
| trt, yr, chickage | 6 | 1953.484 | 4.665 | 0.028 | -970.616 |
| trt, chicks, chickage | 6 | 1953.510 | 4.691 | 0.028 | -970.630 |
| trt x chicks, chickage | 7 | 1954.459 | 5.640 | 0.017 | -970.062 |
| trt x HD, trt x yr, chicks, chickage | 10 | 1955.134 | 6.316 | 0.012 | -967.235 |
| trt x HD, chickage | 7 | 1955.224 | 6.405 | 0.012 | -970.444 |
| trt, chicks, HD, chickage | 7 | 1955.229 | 6.410 | 0.012 | -970.447 |
| trt, HD, yr, chickage | 7 | 1955.233 | 6.414 | 0.012 | -970.449 |
| trt x chicks, trt x HD, trt x yr, chickage | 11 | 1955.233 | 6.414 | 0.012 | -966.217 |
| trt, chicks, yr, chickage | 7 | 1955.566 | 6.747 | 0.010 | -970.615 |
| chickage | 4 | 1955.761 | 6.942 | 0.009 | -973.821 |
| trt x chicks, HD, chickage | 8 | 1956.020 | 7.202 | 0.008 | -969.794 |
| trt x chicks, yr, chickage | 8 | 1956.518 | 7.699 | 0.006 | -970.043 |
| yr, chickage | 5 | 1956.592 | 7.774 | 0.006 | -973.207 |
| trt x HD, yr, chickage | 8 | 1957.320 | 8.502 | 0.004 | -970.444 |
| trt, chicks, HD, yr, chickage | 8 | 1957.324 | 8.506 | 0.004 | -970.446 |
| HD, chickage | 5 | 1957.591 | 8.772 | 0.004 | -973.706 |
| chicks, chickage | 5 | 1957.760 | 8.941 | 0.003 | -973.791 |
| trt x chicks, HD, yr, chickage | 9 | 1958.129 | 9.310 | 0.003 | -969.793 |
| trt x chicks, trt x HD, chickage | 9 | 1958.129 | 9.311 | 0.003 | -969.794 |
| HD, yr, chickage | 6 | 1958.580 | 9.761 | 0.002 | -973.165 |
| chicks, yr, chickage | 6 | 1958.659 | 9.840 | 0.002 | -973.204 |
| trt x HD, chicks, yr, chickage | 9 | 1959.426 | 10.608 | 0.001 | -970.442 |
| trt x chicks, trt x HD, yr, chickage | 10 | 1960.251 | 11.432 | 0.001 | -969.793 |
| chicks, HD, yr, chickage | 7 | 1960.657 | 11.839 | 0.001 | -973.161 |
| none | 3 | 1967.552 | 18.734 | 0.000 | -980.741 |

Fixed effects: trt: treatment (fed or control), yr: year, chicks: number of 7- and 8-day-old nestlings alive in nest at time of measurement, HD: standardized hatching date, chickage: age of chicks when measured (7 or 8 days old), none: intercept-only model.
