# Supplementary material for: The Impact of Increased Food Availability on Reproduction in a Long-Distance Migratory Songbird: Implications for Environmental Change?
Source: PLoS One. 2014 Oct 21;9(10):e111180. doi: 10.1371/journal.pone.0111180 (PMC4205087; doi:10.1371/journal.pone.0111180)
Supplement: Table S10 — Model comparisons for first brood nest survival. All models have Male ID as the random factor. Main effects were included for all interactions. AICc is the corrected Akaike's Information Criterion, ΔAICci is the difference in AICc between model i and the best model and wAICci is the AICc weight of the model. Interactions are indicated by × and include all lower order terms as well (e.g. trt × found represents trt + found + trt × found). (DOCX) [file pone.0111180.s010.docx]

**Table S10. Model comparisons for first brood nest survival.** All models have Male ID as the random factor. Main effects were included for all interactions. AICc is the corrected Akaike’s Information Criterion, ΔAICc*_i_* is the difference in AICc between model *_i_* and the best model and *w*AICc*_i_* is the AICc weight of the model. Interactions are indicated by x and include all lower order terms as well (e.g. trt x found represents trt + found + trt x found).

| **Fixed effects** | **K** | **AICc** | **ΔAICci** | **wAICci** | **Log-likelihood** |
| --- | --- | --- | --- | --- | --- |
| none | 1 | 75.332 | 0.000 | 0.327 | -36.645 |
| trt | 2 | 76.655 | 1.323 | 0.169 | -36.265 |
| found | 2 | 77.221 | 1.889 | 0.127 | -36.548 |
| yr | 3 | 77.457 | 2.125 | 0.113 | -35.602 |
| trt, found | 3 | 78.659 | 3.327 | 0.062 | -36.203 |
| trt, yr | 4 | 79.177 | 3.845 | 0.048 | -35.376 |
| trt x found | 4 | 79.372 | 4.040 | 0.043 | -35.473 |
| found, yr | 4 | 79.637 | 4.305 | 0.038 | -35.606 |
| trt x yr | 6 | 79.940 | 4.608 | 0.033 | -33.513 |
| trt, found, yr | 5 | 81.402 | 6.070 | 0.016 | -35.378 |
| trt x yr, found | 7 | 82.257 | 6.925 | 0.010 | -33.513 |
| trt x found, yr | 6 | 82.257 | 6.925 | 0.010 | -34.672 |
| trt x found, trt x yr | 8 | 83.855 | 8.523 | 0.005 | -33.127 |

Fixed effects: trt: treatment (fed or control), yr: year, date: date found, none: intercept-only model.
