# Supplementary material for: The Impact of Increased Food Availability on Reproduction in a Long-Distance Migratory Songbird: Implications for Environmental Change?
Source: PLoS One. 2014 Oct 21;9(10):e111180. doi: 10.1371/journal.pone.0111180 (PMC4205087; doi:10.1371/journal.pone.0111180)
Supplement: Table S11 — Model comparisons for probability of multiple brooding by males per breeding season. Breeding attempts included first clutches, second clutches (after a successful first clutch) and simultaneous clutches (i.e. polygyny) but did not include re-lays. For the analysis, males either did (1) or did not (0) have multiple broods. AICc is the corrected Akaike's Information Criterion, ΔAICci is the difference in AICc between model i and the best model and wAICci is the AICc weight of the model. Interactions are indicated by × and include all lower order terms as well (e.g. trt × HD represents trt + HD + trt × HD). (DOCX) [file pone.0111180.s011.docx]

**Table S11. Model comparisons for probability of multiple brooding by males per breeding season.** Breeding attempts included first clutches, second clutches (after a successful first clutch) and simultaneous clutches (i.e. polygyny) but did not include re-lays. For the analysis, males either did (1) or did not (0) have multiple broods. AICc is the corrected Akaike’s Information Criterion, ΔAICc*_i_* is the difference in AICc between model *_i_* and the best model and *w*AICc*_i_* is the AICc weight of the model. Interactions are indicated by x and include all lower order terms as well (e.g. trt x HD represents trt + HD + trt x HD).

| **Fixed effects** | **K** | **AICc** | **ΔAICci** | **wAICci** | **Log-likelihood** |
| --- | --- | --- | --- | --- | --- |
| trt, HD | 3 | 50.831 | 0.000 | 0.392 | -22.258 |
| trt x HD | 4 | 51.751 | 0.920 | 0.247 | -21.609 |
| trt, HD, yr | 5 | 53.196 | 2.365 | 0.120 | -21.193 |
| trt x HD, yr | 6 | 54.111 | 3.280 | 0.076 | -20.480 |
| trt | 2 | 54.709 | 3.878 | 0.056 | -25.277 |
| trt x yr, HD | 7 | 55.072 | 4.241 | 0.047 | -19.758 |
| HD | 2 | 57.012 | 6.181 | 0.018 | -26.428 |
| trt x HD, trt x yr | 8 | 57.518 | 6.687 | 0.014 | -19.745 |
| trt, yr | 4 | 57.610 | 6.778 | 0.013 | -24.538 |
| HD, yr | 4 | 57.809 | 6.978 | 0.012 | -24.638 |
| trt x yr | 6 | 60.722 | 9.891 | 0.003 | -23.786 |
| none | 1 | 62.335 | 11.503 | 0.001 | -30.142 |
| yr | 3 | 64.326 | 13.495 | 0.000 | -29.005 |

Fixed effects: trt: treatment (fed or control), HD: hatching date of first brood, none: intercept-only model.
